# Poxvirus under the eyes of electron microscope

**DOI:** 10.1186/s42649-022-00080-3

**Published:** 2022-11-14

**Authors:** Jaekyung Hyun

**Affiliations:** Department of Convergence Medicine, School of Medicine, Pusan National University, Yangsan-si, 50612 Gyeongsangnamdo Republic of Korea

**Keywords:** Poxvirus, Transmission electron microscopy, Virus morphogenesis

## Abstract

Zoonotic poxvirus infections pose significant threat to human health as we have witnessed recent spread of monkeypox. Therefore, insights into molecular mechanism behind poxvirus replication cycle are needed for the development of efficient antiviral strategies. Virion assembly is one of the key steps that determine the fate of replicating poxviruses. However, in-depth understanding of poxvirus assembly is challenging due to the complex nature of multi-step morphogenesis and heterogeneous virion structures. Despite these challenges, decades of research have revealed virion morphologies at various maturation stages, critical protein components and interactions with host cell compartments. Transmission electron microscopy has been employed as an indispensable tool for the examination of virion morphology, and more recently for the structure determination of protein complexes. In this review, we describe some of the major findings in poxvirus morphogenesis and the contributions of continuously advancing electron microscopy techniques.

## Introduction

Recent increase in the number of monkeypox cases around the world is raising a global concern about potential pandemic (Kozlov 2022). After devastating economic and social crisis caused by COVID19 pandemic in the past few years, newly emerging zoonotic viral infection is especially alarming (Mofijur et al. 2021; Palmore and Henderson 2022). Monkeypox virus belongs in a family of poxviruses, *Poxviridae*, that includes variola virus which is a causative agent for infamous smallpox disease that claimed millions of lives throughout the history. Despite complete eradication of smallpox in 1980, zoonotic infections by other poxvirus species and possible bio-terrorism have been continuous threats to human health (Meyer et al. 2020). Although monkeypox has relatively lower infectivity and fatality than smallpox, it has become an endemic in central and western Africa with up to 10% fatality. Occasional human infections by other genera of poxviruses such as orthopoxvirus, molluscipoxvirus and yatapoxvirus emphasize additional possibilities of unexpected zoonotic outbreaks. It is therefore important to understand replication cycle and virulence factors of poxviruses in molecular details, and to develop antiviral strategies.

Poxviruses are enveloped viruses with double-stranded DNA genome with the size ranging from approximately 130 kb (Gunther et al. 2017) to 450 kb (Mekata et al. 2021). Poxviruses infect vertebrates and arthropods, and the viral infections lead to skin lesions that are often mild, but can also be fatal as in the case of smallpox. Poxviruses assemble in the host cytoplasmic compartment called virus factory and exist as various assembly intermediates such as immature virion, mature virion and extracellular enveloped virion. Mature poxviruses typically have ovoid or brick-shaped morphology with length and width ranging from 220 to 450 nm and 140 to 260 nm, respectively. They are one of the largest known animal viruses and often classified as nucleocytoplasmic large DNA viruses (NCLDVs) based on phylogeny and similar replication pathways (Koonin and Yutin 2019). Mature virion is composed of surface membrane with tubular protein decoration, lateral bodies and biconcave core containing nucleoprotein and DNA. The virion may acquire additional membrane from trans-Golgi cisternae or plasma membrane forming wrapped virion and extracellular enveloped virion. While molecular understanding of poxvirus structure and its complex assembly pathway is still limited, most of what we know about virion morphology now is largely owing to transmission electron microscopy studies.

Upon the invention of transmission electron microscope (TEM) in the early 1930s, viruses have been the subjects of extensive investigation because the nanoscopic microbial agents other than bacteria were beyond the achievable resolution range of light microscopes, which were the only instruments available for imaging microbial entities at the time. Some of the earliest observations of viruses by TEM were pioneered by Helmut Ruska, the brother of Ernst Ruska who invented TEM, and date back to late 1930s (Ruska et al. 1939). One of the viruses that were first to be seen using TEM was vaccinia virus (VACV), a prototype poxvirus. While the early observations of VACV only revealed brick-shaped overall morphology of mature virion, in subsequent years TEM analysis have played pivotal roles in understanding poxvirus morphology and replication cycle through examinations of purified virus particles or resin-embedded thin sections of virus-infected cells. In addition, molecular genetics study in combination with immuno-electron microscopy (immuno-EM) allowed for the identification and cellular localization of proteins involved in morphogenesis. More recently, development of cryo-electron microscopy (cryo-EM) and electron tomography (ET) have facilitated molecular understanding of poxviruses as well as the structural insights into replicative machineries of poxviruses. Here we review some of the major findings in poxvirus research, especially focusing on viral morphogenesis of prototypal VACV, aided by TEM studies. We also discuss current limitations and future prospective of structural investigations into poxvirus biology.

### Early characterization of poxvirus morphology

**Fig. 1:**
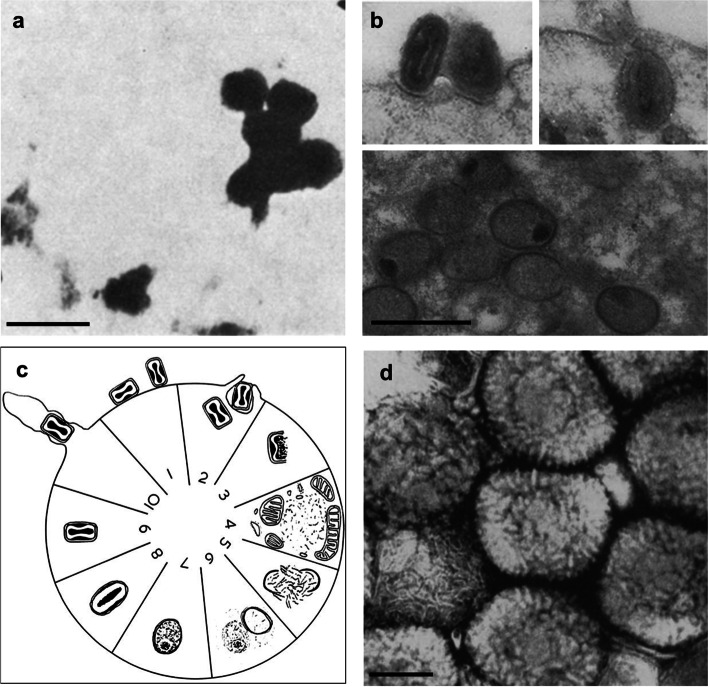
Early TEM examinations of VACV and proposed virion assembly. **a **Brick-shaped morphology of VACV “elementary bodies” first described by Ruska in 1939. **b **Thin-section TEM images of VACV mature virions (top row) and immature virions containing nucleoids (bottom row). **c **VACV assembly cycle as proposed by Dales, showing schematic illustration of virion morphology and post-infection time in hours. **d **TEM image of negatively stained whole VACV mature virion exhibiting characteristic surface tubule decoration. Scale bars are 0.5 μm in (**a**) and (**b**), and 0.1 μm in (**d**). The images are reprinted with permission. Original works are from **a** Ruska et al., Archiv für die gesamte Virusforschung, 1939, doi: 10.1007/BF01243399, **b**, **c **Dales and Siminovitch, The Journal of Biophysical and Biochemical Cytology, 1961, doi: 10.1083/jcb.10.4.475, **d **Dales, The Journal of Cell Biology, 1962, doi: 10.1083/jcb.13.2.303

The earliest TEM examination of VACV mounted on collodion film revealed individual or aggregated “elementary bodies” that are not round or spherical, in consistence with typical brick-shaped poxvirus morphology which was unknown at the time (Ruska et al. 1939) (Fig. 1a). The study was followed by others to reveal that the virion formation in cytoplasm occurs from progressive expansion of crescent-shaped precursors into immature virions, followed by dramatic morphological change into mature virions (Morgan et al. 1954; Wyckoff 1953). Sample preparation technique for clear morphological analysis of biological specimen was lacking until the introduction of methods such as tissue fixation, resin embedment and negative staining in the 1950s (Huxley 1963; Luft 1961; Palade 1952; Watson 1958). Using the improved techniques, observation of detailed morphology of VACV ultrastructure in the context of host cell infection became available (Peters 1956). Moreover, in correlation with virus post-infection time, images of virions entering the host cell and post-entrance were captured in great details (Dales and Siminovitch 1961) (Fig. 1b). Distinctively visible membrane layers surrounding the virions inside the cytoplasm indicated host entrance via phagocytosis, while other virions without the surrounding vesicle suggests other mechanism for direct membrane penetration. After 2 h post-infection, multiple electron-dense foci in the host cytoplasm with associated fibrillar structures started to appear, which coincides with accumulation of genetic materials (Cairns 1960). These so-called “viral factories” are the locations where newly assembled virus particles develop from partially closed membranes into spherical immature virions (IV) containing dense nucleoid. Dramatic morphological changes occur and mature virions (MV) start to appear 6 h post-infection. In contrast to IV that exhibit spherical morphology, MV exhibit ovoid overall morphology with outer membrane, dumbbell-shaped core in the center and the two lateral bodies between the core and the outer membrane. The number and size of virus factories increase subsequently as reflected on maximum virus titer by 24 h post-infection while viruses exiting the host cell are observed as early as 10 h post-infection. The sequence of VACV development was therefore established as summarized Dales and co-workers, as shown in Fig. 1c. In a continued effort, virus particles isolated from VACV-infected cell lysate were examined after negative staining (Dales 1962) from which the virion surface is heavily decorated with numerous short rodlets were observed, giving it a mulberry-like appearance (Fig. 1d).

Despite these great advances in the insights into VACV morphogenesis by TEM, artifacts derived from sample preparation and limited dimensionality in the observations precluded accurate estimation of virion features, leading to controversies over the origin and organization of virion membrane (Dales and Mosbach 1968; Griffiths et al. 2001; Roos et al. 1996; Sodeik et al. 1993), exact virion dimensions (Cyrklaff et al. 2005; Dales and Pogo 1981; Griffiths et al. 2001; Malkin et al. 2003) and the integrity of notable features such as surface tubules (Dales 1962; Dubochet et al. 1994; Westwood et al. 1964). Moreover, lack of the identification of protein components within the virion hampered correlation between the morphogenesis and specific gene products.

### From virion morphology to proteomics

**Fig. 2:**
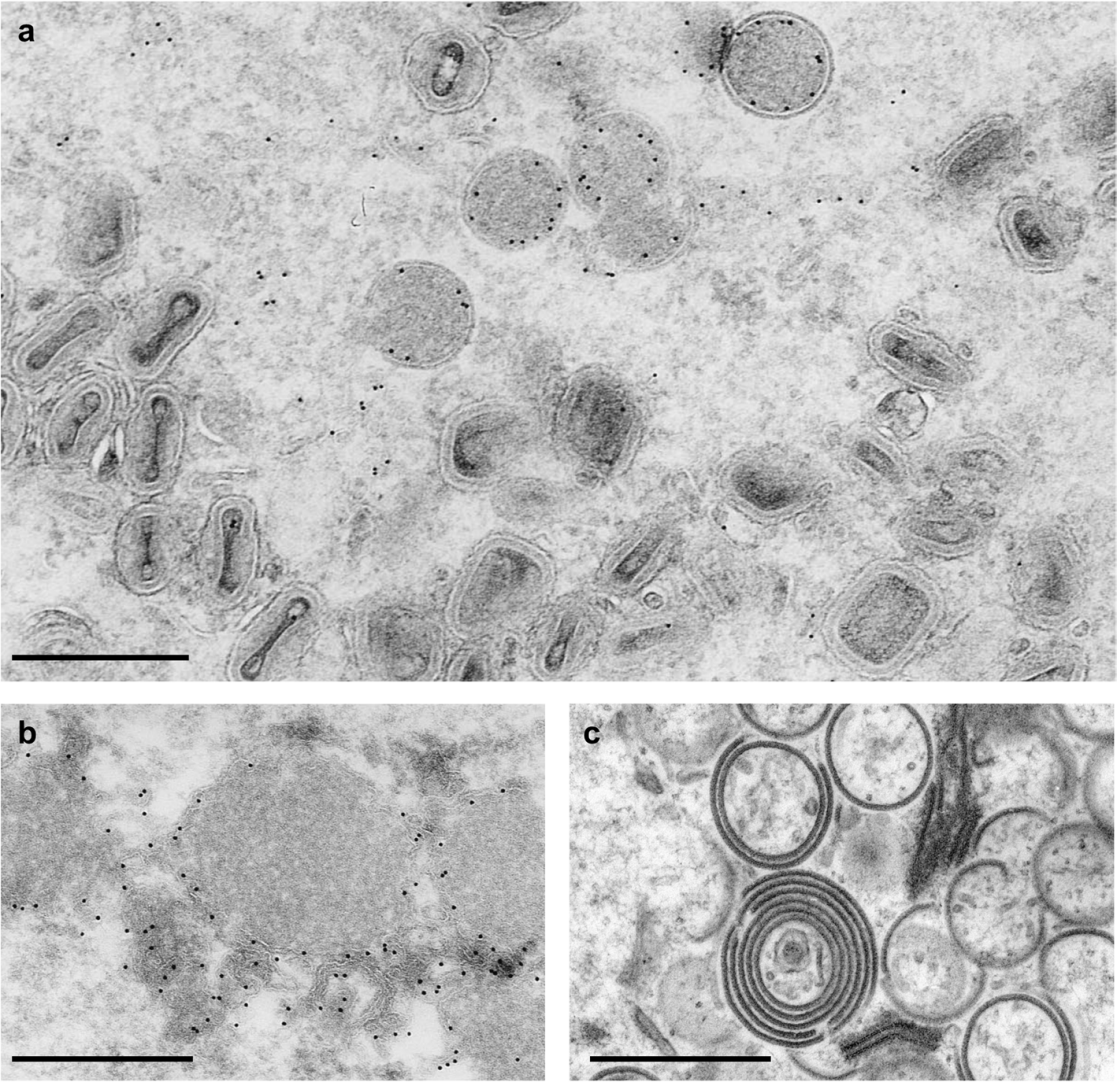
Correlation between virion morphology and protein components. **a **Thin-section TEM image showing immature and mature VACV virions inside host cytoplasm in which specific protein (A14) is identified by immuno-gold labeling. **b **Aberrant virion assembly induced by the addition of assembly inhibitor, rifampicin, showing electron-dense aggregation instead of properly assembled virions. **c **Aberrant virion assembly in the absence of viral protein A30 in which the protein expression was controlled by IPTG induction. Scale bars are 0.5 μm. The images are reprinted with permission, from **a**, **b** Wolffe et al. Journal of Virology, 1996, doi: 10.1128/JVI.70.5.2797-2808.1996, **c **Szajner et al. Journal of Virology, 2001, doi: 10.1128/JVI.75.13.5752-5761.2001.

Identification of gene products that are essential for VACV proliferation has been aided by the studies on conditionally lethal mutants. Given the non-permissive conditions such as high temperature, the specific genes that are mutated becomes highly unstable or non-functional, hence preventing viral replication. The mutant viruses that contains these conditionally non-functional proteins are grown in permissive conditions and the deleterious effects are examined under non-permissive condition. Therefore, particular genes can be “switched off” by raising the incubation temperature for such temperature-sensitive mutants (Condit and Motyczka 1981; Condit and Niles 1990; Dales et al. 1978; Sambrook et al. 1966). Alternatively, a particular gene may be knocked out or engineered in order to control the protein expression in an inducible manner (Zhang and Moss 1991). Complementary TEM analysis of mutant viruses under non-permissive conditions provide excellent method for identifying the genes that are crucial for poxvirus morphogenesis because the absence of critical proteins is reflected on the virus phenotype. Due to distinctive features of VACV assembly at various stages, from crescent precursor to IV and MV, mutations on particular genes can be directly categorized into groups in which how the assembly is affected. One of such examples is a group of temperature-sensitive mutants that results in the loss of protein spicules that decorate the viral membrane of IV (Dales and Mosbach 1968) that resembles morphological defects observed from the addition of antibiotic rifampicin (Moss et al. 1971). The spicule protein was later identified as the product of D13L gene that confers resistance to rifampicin, which was later confirmed to be protein responsible for honeycomb-shaped scaffold on IV membrane that dictates size and shape of the virions (Heuser 2005; Szajner et al. 2005). Using a similar approach, membrane proteins A14 and A17 (Krijnse-Locker et al. 1996; Rodriguez et al. 1997; Unger et al. 2013; Wolffe et al. 1996), and viral membrane assembly proteins (L2, A30.5, A11, A6, H7) were identified as critical proteins required for viral membrane formation and IV assembly (Moss 2015). Another powerful approach to correlate protein function and virion morphology is immuno-gold labelling. Analogous to immunohistochemistry, antibodies conjugated with gold nanoparticles are used to label specific protein on ultrathin sections of virus-infected cells thereby visualizing the localization and distribution of the protein in the context of viral replication (Mohandas and Dales 1995; Sodeik et al. 1993). Immuno-EM frequently accompanies aforementioned conditional mutant studies in order to examine the association of proteins in cellular organelles and assembling virions, and the causal relationship between the mutation and aberrant virion morphology (Fig. 2).

### In the era of Cryo-3DEM

**Fig. 3:**
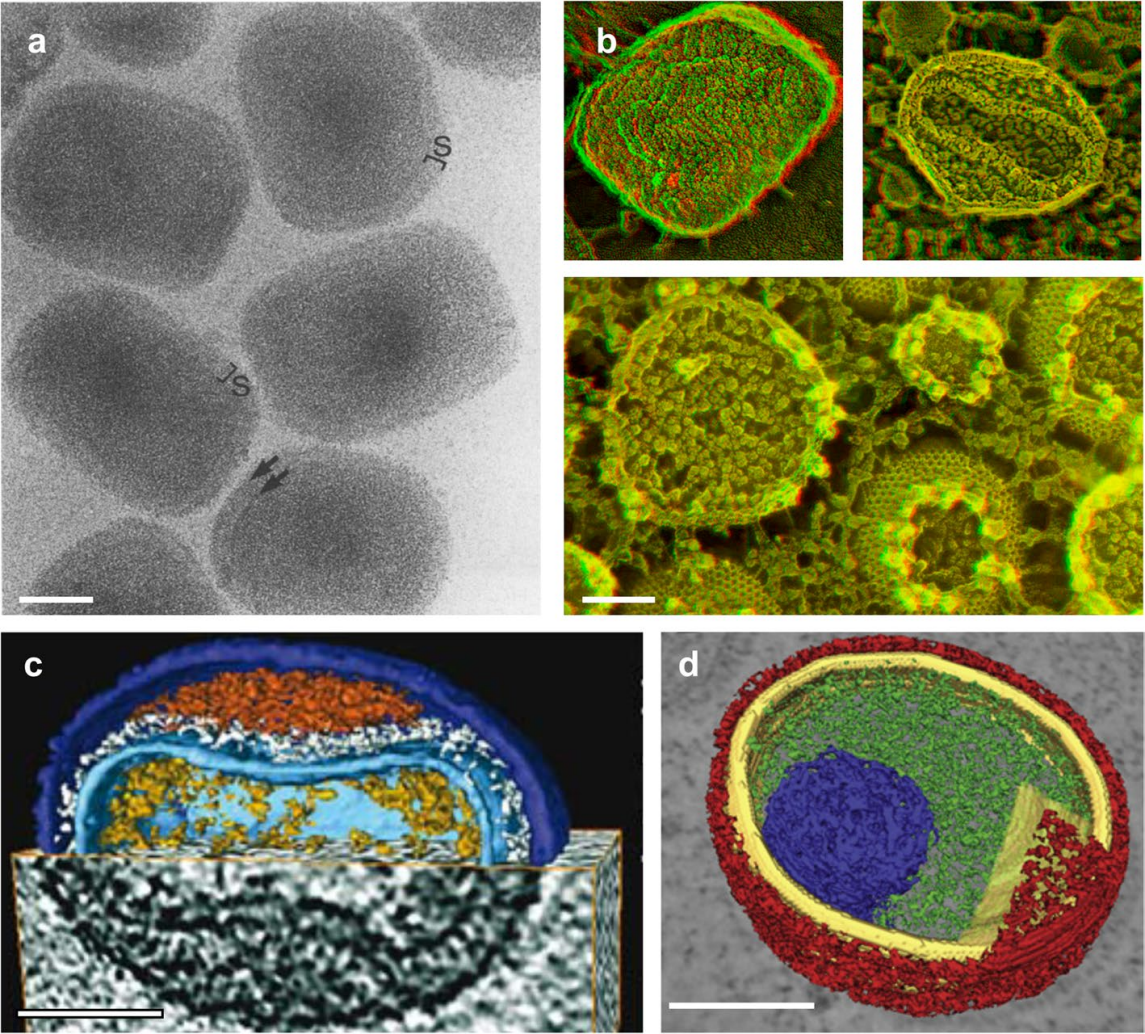
Morphological studies of VACV in the era of cryogenic 3DEM. **a **A cryo-electron micrograph of vitrified VACV mature virion showing surface domain (labeled S) and two membrane boundaries (arrows). **b **3D deep-etch microscopy images showing external and internal morphologies of mature VACV virion (top row) and characteristic honeycomb-like scaffold of immature virion (bottom row). **c **Cryo-electron tomogram of mature VACV virion where top half is shown by surface rendering and bottom half by tomographic section. **d **Surface representation of cryo-electron tomogram of immature VACV virion. Scale bars are 0.1 μm. The images are reprinted with permission, from **a **Dubochet et al. Journal of Virology, 1994, doi: 10.1128/jvi.68.3.1935-1941.1994, **b **Heuser, The Journal of Cell Biology, 2005, doi: 10.1083/jcb.200412169 and Szajner et al., The Journal of Cell Biology, 2005, doi: 10.1083/jcb.200504026, **c **Cyrklaff et al. PLoS One, 2007, doi: 10.1371/journal.pone.0000420, **d **Chlanda et al. Cell Host & Microbe, 2009, doi: 10.1016/j.chom.2009.05.021

Genetic studies, molecular biology and accompanying TEM imaging discovered critical protein components in poxvirus assembly, not to mention the other proteins that render virulence and replicative machineries. However, artifacts in conventional TEM methods and limited resolution have precluded the understanding of exact mechanism behind poxvirus morphogenesis. The method of sample vitrification pioneered by Dubochet and colleagues greatly reduced artifacts derived from chemical fixation and dehydration because cryo-EM allows for the examination of fully hydrated sample in near-native conditions (Dubochet et al. 1982). Early cryo-EM analysis on isolated VACV MV challenged widely-accepted morphology of the virion (Dubochet et al. 1994) (Fig. 3a). The study proposed unambiguous virion dimensions which were found to be from 15 to 40% larger than previous reports. Also, it was concluded that distinct surface tubules that surround viral membrane and dumbbell-shaped core are artifacts due to dehydration and negative staining. However, these claims were later proven to be false based on the evidences provided in other studies (Heuser 2005; Malkin et al. 2003).

In addition to the sample preparation artifacts, major difficulty in morphological analysis of a large and complex virus such as VACV is limited dimensionality of the observation using traditional thin-section approaches. One of the most debated controversies regarding VACV morphogenesis due to this effect is the origin and generation of viral membrane. Initial proposal that single lipid bilayer membrane is produced de novo by Dales and colleagues (Dales and Mosbach 1968) had been challenged by alternative ideas that membrane originates from two apposed lipid bilayers of cisternae, based on the notion that all biological membrane is produced from preexisting membrane (Griffiths et al. 2001; Sodeik et al. 1993). The debate could not be settled due to a number of reasons. Firstly, observations of the number of lipid bilayers in crescent precursor and IV were ambiguous due to sample preparation artifacts. Secondly, visual evidences that demonstrate connection between virial membrane to cisternae were limited, which would require large field of view around the virions in 3D. Thirdly, understating in the involvement of protein components that either hijack cellular membrane or stabilizing hydrophobic edge of singular lipid bilayer was lacking. A thorough morphological characterization by Heuser and colleagues using deep-etch electron microscopy (DEEM), in which sample is frozen without chemical fixation and replica internal structures of fractured virions is examined, providing excellent insights that elucidated some of the unanswered question (Heuser 2005) (Fig. 3b). The study confirmed that crescent and IV are indeed composed of single membrane lipid bilayer, but with no obvious membranous connection to cellular compartments. One of the most notable features was confluent honeycomb-shaped scaffold made of protein D13 that surrounds IV membrane, which was proposed to be a key component that stabilize viral membrane. Cryo-electron tomography (cryo-ET) study by others provided direct 3D views of authentic MV and virion disassembly upon host cell binding (Cyrklaff et al. 2005, 2007). In all these 3D electron microscopy (3DEM) approaches, classical morphology of the virion such as surface tubules and biconcave core with palisade layer were shown to be real, and not attributed to artifacts of traditional methods. Advances in cryo-ET, especially for the method of sectioning VACV-infected cells that are otherwise too thick to transmit electron beam, further provided insights into initial stage of virion assembly. Studies by Chlanda used cryo-EM of vitreous sections (CEMOVIS) and reaffirmed that viral membrane is composed of single lipid bilayer and covered with hexagonal lattice of D13 protein (Chlanda et al. 2009). Also, major viral membrane A14 was labeled using immuno-EM to trace connectivity with crescent, leading to the observation that D13-studded crescents are connected to uncoated, open membrane sheet or closed membrane compartments. The study proposed closed membrane structures originated from endoplasmic reticulum are ruptured, and stabilized by inverted conical lipids and specialized viral membrane proteins. Open membrane is shaped into crescent in association with partially assembled D13 scaffold in which additional membranes are fused to assembling viral membrane. Another study by Chichon and co-workers employed freeze-substitution, a method that fixates the sample under low temperature and thereby minimizing dehydration-induced artifacts, and chracterized viral membrane re-organization from IV, incorporation of nucleoid and into MV (Chichon et al. 2009).

Following the new proposals that specialized proteins play critical role in virion morphogenesis, numerous conditionally lethal mutants were produced and subjected to TEM examinations of assembly defects. For example, cryo-ET examinations of deletion mutants of viral membrane assembly proteins (VMAPs), a group of proteins including L2, A30.5, A11, A6 and H7, demonstrated that one or more VMAPs interact for ER membrane hijacking and the separation of viral membrane from its origin (Suarez et al. 2017; Weisberg et al. 2017). Recent study proposed H7 is directly relevant in hexameric organization of D13 scaffold protein, suggesting complex interactions among VMAPs, D13, A17 and A14 (Tonnemacher et al. 2022).

### From virion morphology to protein structures

Detailed molecular mechanism behind virus replication is often aided by determination of protein structures. To date, 111 VACV protein structures are available in Protein Data Bank. Determined structures include proteins that are involved in viral entrance and release, DNA replication, host immune modulation and other replicative machineries. The structures were mostly determined by x-ray crystallography whereas recent technical breakthroughs in cryo-EM single particle analysis facilitated structural insights into large complexes such as virus-encoded multi-subunit RNA polymerase complex (Grimm et al. 2019, 2021).

**Fig. 4:**
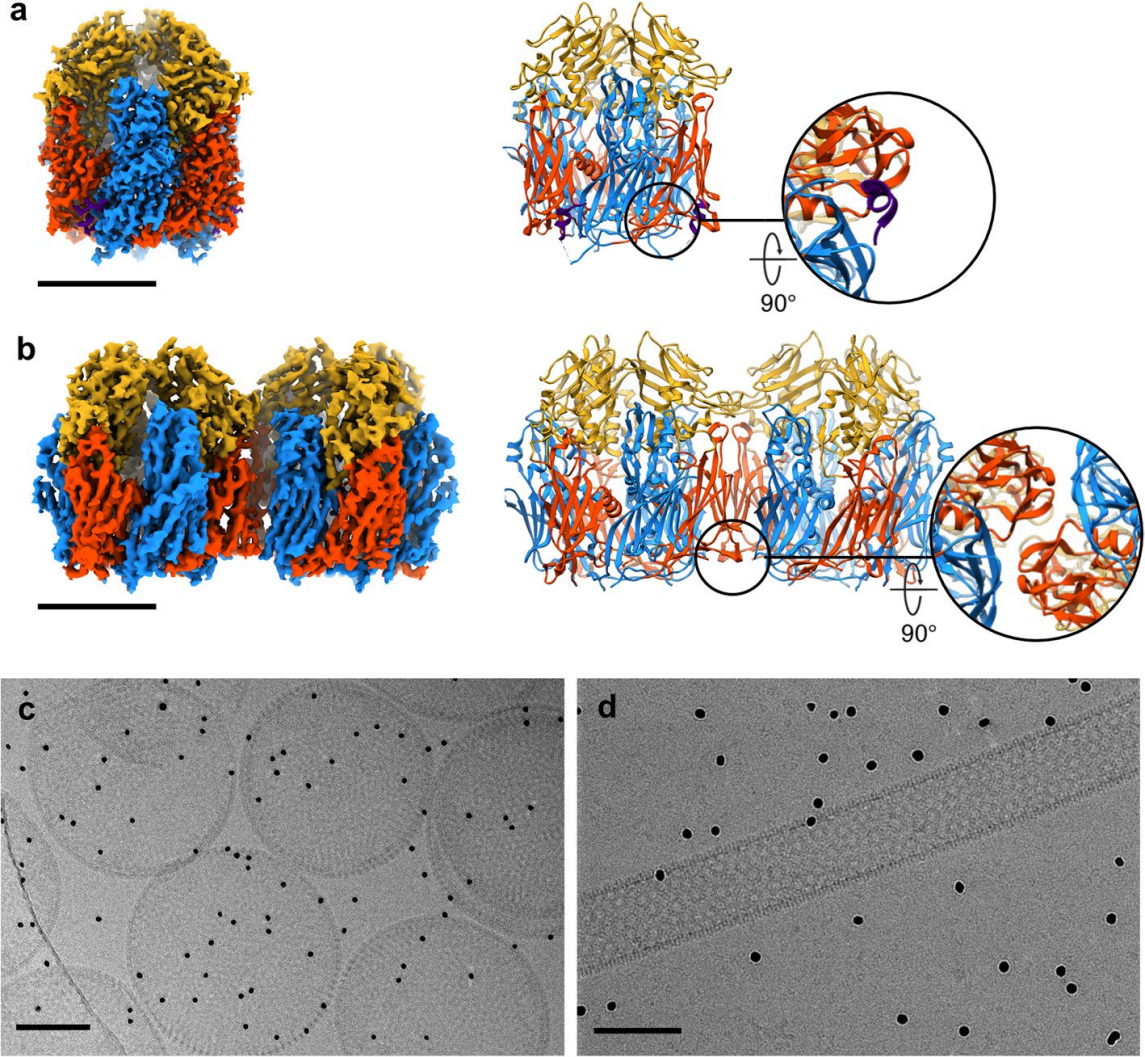
Cryo-EM structure of VACV D13 and its assembly. **a **Single particle cryo-EM 3D reconstruction of VACV scaffolding protein D13 in its native trimeric form (left) and the ribbon representation of its atomic model with a close-up view of N-terminal tail α-helix (right). **b **Single particle cryo-EM 3D reconstruction of D13 trimer doublet (left) and the ribbon representation of its atomic model with a close-up view of the inert-trimer interface where N-terminal tail α-helix is missing (right). The reconstructions and the models are colored according to domains in D13 monomer (blue: N-terminal jellyroll domain, red: C-terminal jellyroll domain, yellow: head domain, purple: N-terminal tail α-helix helix). **c **Spherical and **d **tubular assemblies produced in vitro under low salt condition when the N-terminal tail α-helix is dislocated or truncated, suggesting that the disposition of the helix triggers D13 self-assembly into IV scaffold. Scale bars are 5 nm in (**a**) and (**b**), and 100 nm in (**c**) and (**d**)

While the structures of proteins involved in poxvirus morphogenesis are largely unknown, the structure of scaffold protein D13 has been relatively well-characterized. First two crystal structures of D13 trimer have shown double jellyroll secondary structure motif and addressed common viral lineage with NCLDVs which share similarly folded major capsid proteins (Bahar et al. 2011; Hyun et al. 2011). In addition to atomic structures, organization of honeycomb-shaped scaffold lattices were demonstrated by in vitro assembly of orthopoxvirus (orf virus) scaffold protein and for VACV D13, at moderate resolutions (Hyun et al. 2007, 2011). Long-awaited mechanism of rifampicin on the inhibitory effect of IV formation was addressed by crystal structure of D13 in complex with rifampicin and its derivatives, indicating the interaction between D13 and its binding partner A17 is competitively interfered by rifampicin on the same binding site (Garriga et al. 2018). In a recent cryo-EM study of D13 and its assembly intermediates produced in vitro, it was proposed that inter-timer interaction is initiated by displacement of loosely held N-terminal α-helix which leads to electrostatic interaction at two major interfaces between the trimers. It was also demonstrated that the torsion between the interface induces continuous curvature that allows for the assembly into spherical IV scaffold (Hyun et al. 2022) (Fig. 4). The crystal structure of H7 protein, one of the VMAPs that are critical for viral membrane assembly, showed novel fold with phosphoinositide binding site and the structure-guided mutations to the binding site inhibit VACV replication (Kolli et al. 2015). However, the cooperativity among VMAPs and with other critical proteins are yet to be elucidated, for which structure of the functional complexes need to be determined in the context of authentic virion assembly.

## Conclusion

In this review we have described the advancements in the understating of poxvirus morphogenesis aided by technical breakthroughs in TEM. More comprehensive reviews are available on VACV in general (Condit et al. 2006; Damon 2013; Moss 2013), membrane biogenesis (Moss 2015, 2018), modulations to cellular pathways (Lant and Maluquer de Motes, 2021; Suraweera et al. 2020). Despite the technical advances, complexity of poxvirus structure and its assembly pathway, and limited in situ visualization of the virus in the host cell make the analysis particularly challenging. Development of correlative light-electron microscopy, together with focused ion beam-based specimen milling of vitrified cells and cryo-ET, promises a new avenue for studying the interactions among viral proteins that are associated with morphogenesis. Moreover, recently emerging subtomogram averaging technique would be an excellent approach for bridging the gap between the low-resolution tomogram and structural insights (Schur 2019). However, method to accurately label small membrane proteins such as VMAPs in the cells and subtomogram averaging of such voxels for structure determination remain to be hurdles to overcome. Alternative to in situ structural biology is the development of in vitro systems that can closely mimic authentic virion. As in the cases of capsid and Gag polyprotein structures of HIV-1(Bharat et al. 2014; Schur et al. 2016; Zhao et al. 2013), virus-like particles and in vitro nucleocapsid assemblies of Ebola virus (Bharat et al. 2012; Sugita et al. 2018; Wan et al. 2017, 2020) and VACV D13 assembly intermediates (Hyun et al. 2022), in vitro systems allow for mass-production of objects that can be studied analogous to authentic virus assembly. Such divide-and-conquer strategies in parallel with complementary in situ visualization will lead to new insights.

## Data Availability

Not applicable.

## Abbreviations

CEMOVIS: Cryo-electron microscopy of vitreous sections
Cryo-EM: Cryo-electron microscopy
DEEM: Deep etch electron microscopy
ET: Electron tomography
Immuno-EM: Immuno-electron microscopy
IV: Immature virion
MV: Mature virion
NCLDV: Nucleocytoplasmic large DNA virus
TEM: Transmission electron microscope
VACV: Vaccinia virus
VMAP: Viral membrane
